# The Beneficial Atrial Septal Defect Shunt in Hypertrophic Cardiomyopathy—When Closure Is Not the Answer

**DOI:** 10.1016/j.jscai.2024.102281

**Published:** 2024-09-02

**Authors:** Mohammad Alnoor, Ahmed Deniwar, Sherzana Sunderji

**Affiliations:** Division of Pediatric Cardiology, University of California–Davis, Davis, California

**Keywords:** atrial septal defect, diastolic dysfunction, hypertrophic cardiomyopathy

## Introduction

Hypertrophic cardiomyopathy (HCM) affects 0.2% of the general population, with lower frequency in the pediatric population, albeit with higher incidence of associated cardiac defects.^1^ An atrial septal defect (ASD) can result in a left-to-right shunt that decreases preload to the left ventricle (LV). Eliminating this shunt can be favorable in a hypertrophied LV, allowing for improved filling and decreasing outflow obstruction. In diastolic dysfunction, an ASD can serve as a “pop-off” to decompress the LV, with closure resulting in pulmonary edema and activity intolerance.^2^ In large ASD, the left atrial pressure may also be reassuring as it equates with the right atrial pressure; therefore, hemodynamic assessment can be beneficial in procedural decision making.

## Case report

**Figure 1:**
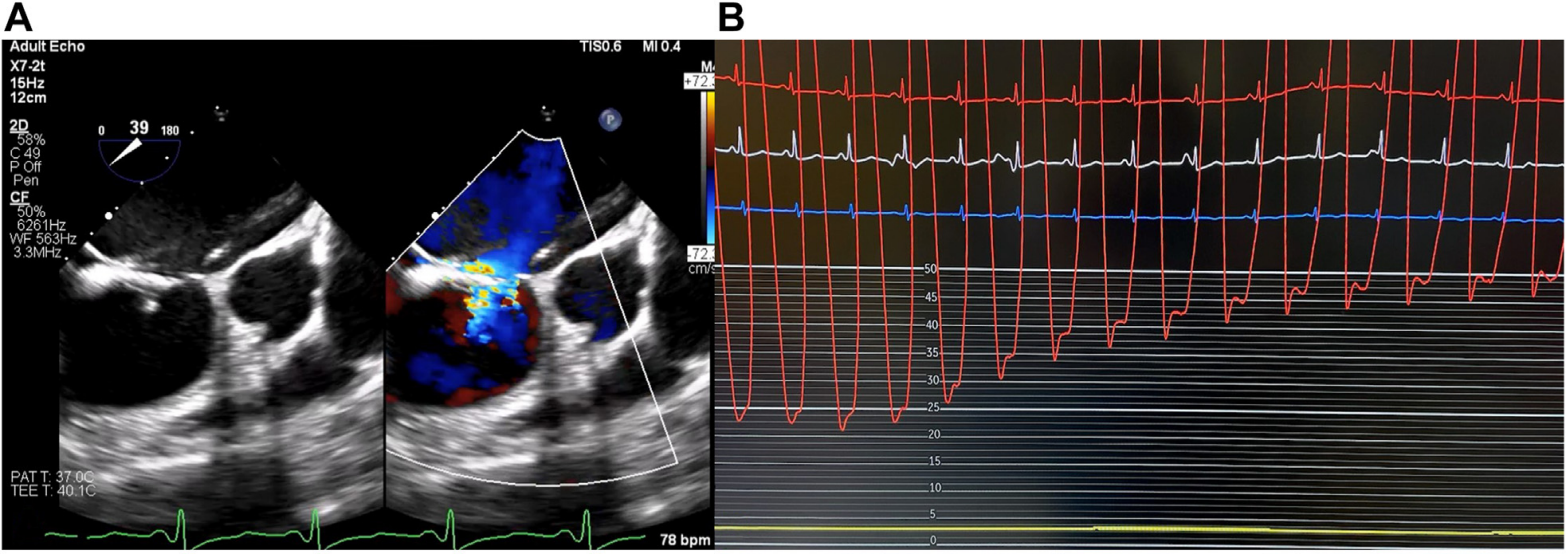
(**A**) The transesophageal echocardiographic image of partial balloon occlusion of the atrial septal defect, with residual flow around the balloon. (**B**) The increase in left ventricular end-diastolic pressure with partial balloon occlusion.

A 26-year-old woman was diagnosed with HCM following workup initiated after diagnosis in a first-degree relative. Cardiac magnetic resonance imaging showed reverse curve HCM with severe septal hypertrophy, a 7.0 × 10.0 mm ASD with moderate right atrial enlargement. She was asymptomatic but, given the heart dilation, was referred for ASD closure. Baseline hemodynamics revealed the following: Qp:Qs, 2; mean pulmonary artery pressure, 29 mm Hg; mean right atrial pressure, 14 mm Hg; mean left atrial pressure, 20 mm Hg; and left ventricular end-diastolic pressure (LVEDP), 20 mm Hg. Balloon occlusion testing of the ASD was performed simultaneously with a catheter in the LV. The LVEDP acutely increased to 50 mm Hg. Partial balloon testing was performed to determine candidacy for a fenestrated occluder, but LVEDP similarly increased to 50 mm Hg (Figure 1). Given persistent diastolic pressure elevation with only partial balloon occlusion, the ASD was left patent.

## Discussion

While transcatheter ASD closure has become more common, severe diastolic dysfunction is likely a contraindication for complete ASD closure, with thorough hemodynamic assessment required before device placement, particularly in high-risk patients. Routine ASD closure requires venous access for the entirety of the procedure. We elected to place additional arterial access to provide continuous LVEDP monitoring, revealing significant diastolic pressure elevation even with partial balloon occlusion, likely due to increased volume load on a noncompliant ventricle.

Published case series describing ASD closure in HCM and/or diastolic dysfunction,^2^^,^^3^ describe most operators electing placement of fenestrated ASD devices. In our patient, the small ASD was beneficial in the setting of the diastolic dysfunction, contributing to the lack of symptoms. This case highlights the importance of a high index of suspicion in at-risk populations and the value of thorough hemodynamic assessment during transcatheter ASD closure.

## Pearls in Hemodynamics

• Small ASDs can provide a beneficial pop-off in patients with hypertrophic cardiomyopathy and diastolic dysfunction
• Balloon test occlusion of an ASD with simultaneous measurement of the LVEDP can be valuable in procedural decision making
• A high index of suspicion and thorough hemodynamic assessment should be obtained before ASD closure in at-risk populations
